# First records of *Triatoma rubrofasciata* (De Geer, 1773) (Hemiptera, Reduviidae) in Foshan, Guangdong Province, Southern China

**DOI:** 10.1186/s40249-017-0342-y

**Published:** 2017-08-15

**Authors:** Qin Liu, Yun-Hai Guo, Yi Zhang, Zheng-Bin Zhou, Liang-Liang Zhang, Dan Zhu, Xiao-Nong Zhou

**Affiliations:** National Institute of Parasitic Diseases, Chinese Center for Disease Control and Prevention, Key Laboratory of Parasite and Vector Biology, Ministry of Health, WHO Collaborating Center for Tropical Diseases, Shanghai, 200025 People’s Republic of China

**Keywords:** Triatominae, *Triatoma rubrofasciata*, Foshan, Southern China

## Abstract

**Background:**

Triatomines, also known as kissing bugs, which are found throughout the world and especially in Latin America, are well known natural vectors that transmit American trypanosomiasis, also called Chagas disease. In China, the presence of two species of *Triatoma* (*Triatoma rubrofasciata* and *T. sinica*) was recorded in the past. Due to the growing population and the increasing risk of the global spread of Chagas disease, triatomines became a potential public health nuisance, and in 2016, we started monitoring triatomine activities in southern China.

**Methods:**

Triatomine specimens were collected by the National Institute of Parasitic Diseases, Chinese Center for Disease Control and Prevention, and identified by their morphological characteristics under a dissecting microscope. In addition to morphological analysis, the genomic DNA of the specimens was extracted, and the mitochondrial 16S rRNA, the cytochrome b (CytB) gene and the nuclear ribosomal 28S rRNA gene were PCR-amplified to analyze and confirm the species genetically.

**Results:**

One female adult insect and one male adult insect were collected in a dwelling in the rural area of Shunde County, Foshan City, Guangdong Province, China (22°42′44.63″N, 113°08′45.34″E). The results from the morphological and genetic analyses indicated that these triatomines were *T. rubrofasciata*.

**Conclusions:**

This is the first time that the occurrence of *T. rubrofasciata* has been confirmed in Foshan City, Guangdong Province in southern China. Further studies are needed to reach a clearer understanding of the ecology of this species of triatomine, since it has been found to be naturally infected by *Trypanosoma cruzi* and *T. conorhini* and there is evidence of its domiciliation capabilities.

**Electronic supplementary material:**

The online version of this article (doi:10.1186/s40249-017-0342-y) contains supplementary material, which is available to authorized users.

## Multilingual abstracts

Please see Additional file 1 for translations of the abstract into the five official working languages of the United Nations.

## Background

Triatomines, also known as kissing bugs, are bloodsucking insects of the family Reduviidae and subfamily Triatominae, which are found throughout the world, especially in Latin America [1, 2]. A total of 150 species of Triatominae belonging to 18 genera currently described worldwide are potential vectors that can transmit American trypanosomiasis, also called Chagas disease, which is caused by *Trypanosoma cruzi* and is considered the fourth most transmitted disease after malaria, tuberculosis, and schistosomiasis by the World Bank and World Health Organization (WHO) [3, 4]. Among the Triatominae species, *Triatoma rubrofasciata* is recorded to be the most widely distributed worldwide, extending geographically from the United States of North America (Florida, Hawaii) to Central America (Mexico) and Latin America (Argentina-Buenos Aires, Brazil-Atlantic coastal areas, Cuba, and most other Caribbean islands, French Guiana, Suriname, and Venezuela), from coastal regions of Africa (Angola, Congo-Katanga, Guinea-Conakry, Seychelles, Sierra Leone, South Africa, Tanzania, Reunion, and New Guinea) to the Middle East (Saudi Arabia), and from south Asia-western Pacific (India-Tamil Nadu, China, Indonesia, Malaysia, Sir Lanka, Singapura, Japan, Philippines, Taiwan, Thailand-Bangkok, Vietnam, Andaman Islands, Tonga, Burma-Myanmar, Cambodia, Carolina Islands, Comoros Islands, Madagascar, Mauritius, Rodriguez Islands, Sri Lanka, Singapore, and Seychelles) to the Atlantic Ocean (Azores) [5–8].

In recent decades, Chagas disease has become a global health issue and has attracted much more attention than in the past [3, 9]. Due to growing population movements, important epidemiological changes have occurred, and the disease has now spread to non-endemic countries. At present, Chagas disease has been diagnosed in several non-endemic countries, such as Canada, the United States of America, Australia, New Zealand, and Japan, as well as in Europe [10–12]. Fortunately, there has not yet been any record of Chagas disease in China, but the presence of two species of *Triatoma* (*T. rubrofasciata* De Geer and *T. sinica* Hsiao) were recorded over three decades ago [13]. In the last thirty years, there has been little research on triatomines. Since triatomines are a potential public health nuisance, we started to monitor triatomines in 2016 in southern China.

## Methods

In this study, we discovered the presence of triatomines for the first time in November 2016. One living female adult and one dead male adult were collected in a dwelling in the rural area of Shunde County, Foshan City, Guangdong Province, China (22°42′44.63″N, 113°08′45.34″E) on 10th November 2016. The specimens were discovered inside a masonry structure with two floors. On the first floor, there was a pharmacy shop with wooden ceilings and many wooden shelves containing hundreds of Chinese herbal medicines, mainly from Guangzhou Chinese herbal medicine market, as well as hundreds of western medicines. On the second floor, there was a living-room with two wooden beds and lots of clothes piled in the aisle. The resident reported that 5 triatomine insects were found from September to early November, 2016, including the aforementioned female and male adult specimens. One blood-sucking nymph was found in a child’s quilt, another nymph was found under the corner of a board on the second floor, and a female adult was found in the pharmacy on the first floor. Behind the house was a small yard with low brush (*Murraya paniculata*) and a tall mango tree that extended to the vent of the second floor. On the mango tree, there were some shield bugs and molts. Behind the yard was a small pond that has been filled and built into a kindergarten. Behind the kindergarten, there was an orchard with many banana threes and some palm trees.

The morphological identification of the triatomines was conducted in the Key Laboratory of Parasite and Vector Biology of the National Institute of Parasitic Diseases, Chinese Center for Disease Control and Prevention and referred to Xiao et al. and the website of Centers for Disease Control and Prevention of the United States [13, 14]. Genomic DNA was extracted from 3 legs of the triatomines using the DNeasy Blood & Tissue Kit (Qiagen, German), according to the manufacturer’s recommendations. The mitochondrial 16S rRNA and the cytochrome b (CytB) gene were PCR-amplified using the primers 16S–F (5′-CGCCTGTTTATCAAAAACAT-3′), 16S–R (5′-CTCCGGTTTGAACTCAGATCA-3′) CYTB7432 (5′-GGACGWGGWATTTATTATGGATC-3′), and CYTB7433 (5′-GCWCCAATTCARGTTARTAA-3′) [15, 16]. The nuclear ribosomal 28S rRNA gene was PCR-amplified using the primers 28S–F (5′-GCGAGTCGTGTTGCTTGATAGTGCAG-3′) and 28S–R (5′-TTGGTCCGTGTTTCAAGACGGG-3′) [17]. PCR reactions were conducted in a final volume of 25 μl using 30 ng of DNA template, 2 × *Taq* PCR Master Mix (TianGen, China), and 0.4 μM of each primer. The fragments were amplified with the following thermal cycling conditions: 95 °C for 3 min; 35 cycles of 94 °C for 30 s, 55 °C for 30 s, and 72 °C for 60 s; and 72 °C for 10 min. The target DNA fragments were inserted into pGEM-T easy vectors (Promega), and the plasmids were transformed into *E. coli* DH5ɑ and sequenced (Shanghai Dingan Biotechnology Ltd., Co.).

The sequences were then submitted to the National Center for Biotechnology Information (NCBI) GenBank, and the genetic distance matrix was calculated between the molecular data generated in this study and the data deposited in GenBank from other countries/localities to evaluate the polymorphism of *T. rubrofasciata*. The 16S rRNA sequence of this triatomine was matched with the corresponding sequences of representative species using Clustal 1.8, which were edited manually using BioEdit Sequence Alignment Editor (version 7.0.9.0.; Carlsbad, CA. USA). The phylogenetic trees were constructed using the neighbor-joining method by MEGA6.0 (Molecular Evolutionary Genetics Analysis Version 6.0) [18]. Distance and maximum likelihood models were applied using 1000 bootstrap replicates per tree for each method. The 16S rRNA gene sequence of *Stenopodainae* sp. (JQ897844) was included in the trees as an outgroup.

## Results

**Fig. 1:**
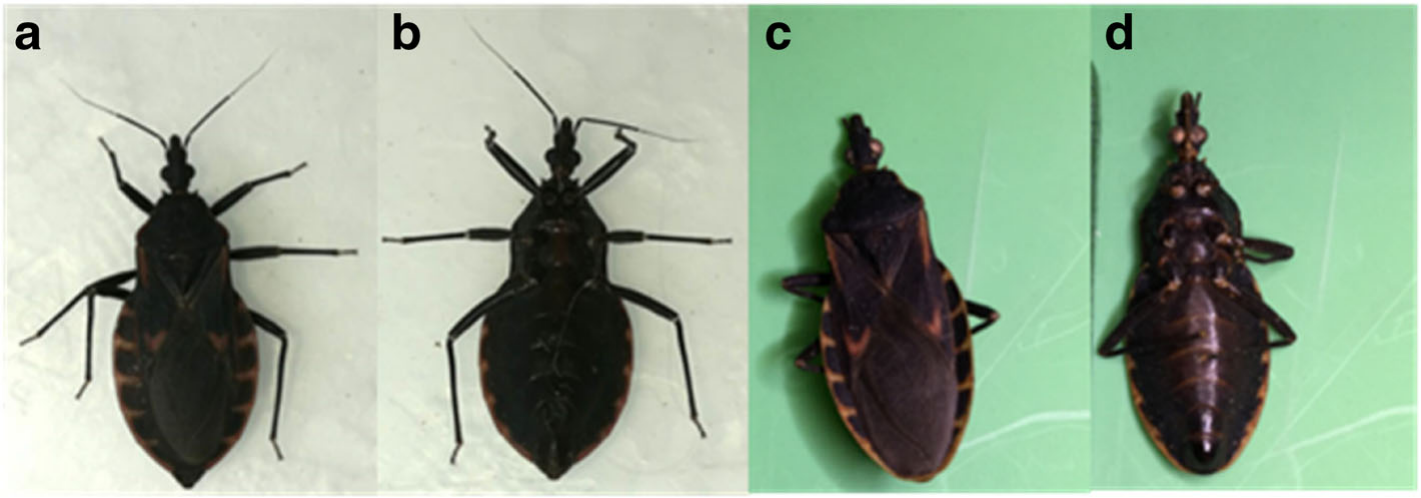
**a** and **b**, Dorsal and Ventral views of female *T. rubrofasciata*; **c** and **d**, Dorsal and Ventral views of male *T. rubrofasciata* specimens found in Shunde, Foshan, Guangdong Province

**Fig. 2:**
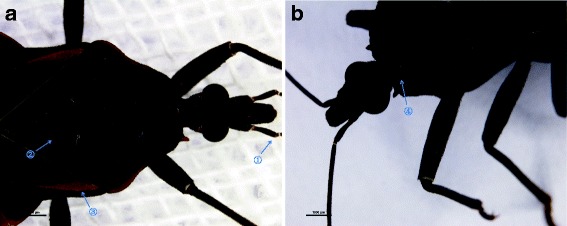
The characterstics of *T. rubrofasciata*. **a** ① 1st antennal segment surpasses tip of head; ② Scutellum broad, triangular to tip; ③ Orange-red margin on sides of pronotum; **b** ④ Mouthpart is straight, and short hairs on mouthparts become progressively longer towards tip

The triatomines were characterized as *T. rubrofasciata* by morphological characteristics (Figs. 1 and 2). The female and the male had lengths of 24 mm and 22 mm, respectively. The head and pronotum were densely covered with small particles, and the head was slightly longer than the pronotum; the head of the female was 3.9 mm, and the pronotum was 3.6 mm. On the female’s body, the abdomen had an orange-red margin on the outer edge, extending horizontally between segments, as well as orange-red on the margins of the pronotum, the 3rd and 4th segment of antennae, margin of the front corner, and the vertical lines and central spot of septum. Its head was 1.1 mm wide with a total of 4 segments of antennae, and the 1st antennal segment surpassed the tip of the head (Fig. 2a). The lengths of each antennae were 1.1 mm, 3.6 mm, 2.3 mm, and 1.9 mm. Its chest width was 2.2 mm to 5.7 mm, characterized by a triangular scutellum with middle rugose and a short and sharp apex. The chest length was 2.6 mm (Fig. 2a). The wing length was 14 mm, and the wing did not extend to the end of the abdomen. The abdomen width was 8.8 mm and was flattened longitudinally underneath. The mouthpart was straight, and short hairs on the mouthpart became progressively longer towards the tip (Fig. 2b).

**Fig. 3:**
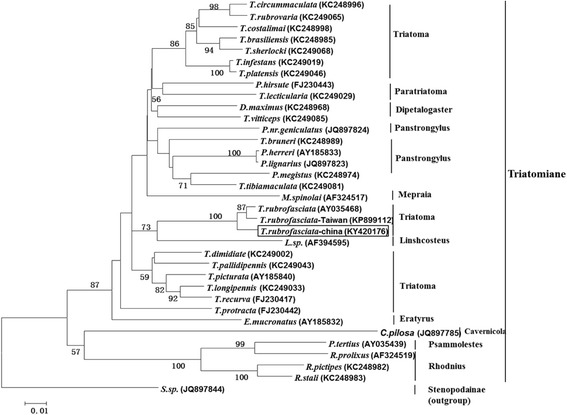
Phylogenetic tree showing the relationships between the identified Triatominae and other species. The relationships were determined using the 16S rRNA gene sequences by neighbor-joining with 1000 bootstrap resamplings. *T. rubrofasciata*-China identified in this study (KY420176) is indicated in the square frame

The sequences obtained from the mitochondrial 16S rRNA, the CytB gene and the nuclear ribosomal 28S rRNA were submitted to GenBank under the accession numbers KY420176, KY420178, and KY420177, respectively. For the polymorphism analysis, 386 bp from 16S rRNA and 651 bp from CytB were obtained, without insertions and deletions. For the 16S rRNA sequences of *T. rubrofasciata*, Chinese species (accession numbers KY420176, KP899112 and AY035668) and Vietnamese species (accession numbers HQ337018 and HQ337019) were used for the analysis. Chinese (KY420176) and Chinese-Taiwanese species (KP899112) individuals showed no differences, and the genetic distance between them was 0.0%. Only one transversion site differed between China (KY420176) and Vietnam (HQ337019), and the genetic distance between them was 0.3%. Five transversion sites differed between China (KY420176) and China (AY035468), and the genetic distance between them was 1.3%. Seven transversion sites and one transition site differed between Chinese (KY420176) and Vietnamese (HQ337018) specimens, and the genetic distance between them was 2.1%. Meanwhile, eight transversion sites and one transition site differed between the Vietnamese samples (HQ 337018 and HQ337019), and the genetic distance was 2.3%. The mean genetic distance between the Chinese and Vietnamese sequences was 3.1%, with 12 variable sites. The CytB sequences of the Vietnamese specimens (KR 632555 and KR 632556) were identical, while the genetic distance between two individuals from Brazil (KR632553 and KR632554) was 0.15%, with only one differing transversion site, and 0.6% for the two Chinese specimens (KY420178 and KP899111) due to 4 differing transversion sites. The mean genetic distance was 0.8% between Chinese and Vietnamese sequences with 5 variable sites, 1.0% between Chinese and Brazil with 7 variable sites, and 0.15% between Vietnamese and Brazilian sequences with 1 variable site. For the nuclear 28S rDNA, the 678 bp sequences obtained (accession numbers KR632546–KR632548, and KY420177) were manually edited to remove all ambiguously aligned nucleotide positions, as is recommended for rDNA genes [19]. The Chinese individual (accession number KY420177) and the two Vietnamese individuals (accession numbers KR632547 and KR632548) showed no differences, and only one variable site differentiated them from the Brazilian samples (accession number KR632546), while the genetic distance was 0.0% between the Chinese and Vietnamese *T. rubrofasciata* samples and 0.1% from the Brazilian sample. Phylogenetic analyses of the 16S rRNA gene sequences of Triatominae demonstrated that the heredity distance of the 16S rRNA gene (AY420176) (in the square frame) and *T. rubrofasciata* 16S ribosomal RNA gene from the Brazilian strain (AY035468) and the Taiwanese strain (KP899112) were in the same clade, and they were close to its sister group *Linshcosteus* sp. (AF394595). These results were also confirmed by molecular techniques, showing that the *Triatoma sp.* from Foshan, Guangdong Province, China was *T. rubrofasciata* (Fig. 3).

## Discussion

This is the first record of the presence of triatomine species in Foshan City, southern China. In this study, both nymphs and adult triatomines were collected, and it is believed that the insects may have resided in the aforementioned house. However, for our epidemiological investigation, in the survey questionnaire, no resident reported that they had seen this species of insect before. A total of 50 Noireau traps were placed around the house and the orchard for three successive nights, and no triatomines were trapped. Therefore, it was difficult to conclude whether the insects were attracted by light from the residence or transported from the Guangdong Chinese herbal medicine market through the imported Chinese herbal medicine.

Both the morphological and molecular evidence indicated that this triatomine was *T. rubrofasciata*. In addition to these taxonomic tools, *T. rubrofasciata* could also be easily identified by cytogenetic analysis (simple and low-cost techniques, but which rely on live triatomines) [20, 21]. This will be suggested for species recognition in future entomological surveys in China. As we know, *T. rubrofasciata* has been confirmed to transmit at least two parasites: *T. cruzi* in Latin America and *T. conorhini* worldwide [5]. At present, *T. cruzi* is found exclusively in the migrating from Latin America. The confirmation of the occurrence of this species in southern China brings a potential risk for transmission of Chagas disease in southern China. By contrast, *T. conorhini* has been recorded in the intestine of *T. rubrofasciata* in many locations in the world [5]. Experiments showed that Asian monkeys were susceptible to this parasite, and it was recorded that the parasite could be opportunistic [5, 22]. Thus, further studies are needed to better understand host-parasite relationships of triatomine and to evaluate the transmission risk of the trypanosomiasis in this region.

As we know, the vast majority of species (95%) of triatomines are limited to the new world; there were only a few species belonging to the genus *Triatoma* and its sister group *Linshcosteus* distributed in the old world [14, 23]. Among them, *T. rubrofasciata* is known to currently be widely distributed [8]. In the present study, the phylogenetic tree results showed the strains of *T. rubrofasciata* and its sister group *Linshcosteus* sp. in the same clade (Fig. 3); these strains belonged to an old world clade and showed monophyly [24]. The current consensus is that the Triatominae have relatively recent origins in the Americas and that the old world species represent derivatives from an American form [23]. To date, the question of the mode of dispersal of *T. rubrofasciata* to the old world and to other continents is natural and unanswered [24]. One hypothesis is that *T. rubrofasciata* was accidentally carried on ships by infested mice from Latin America to elsewhere [23, 24]. Thus, more data was needed to add to the analysis of the derivatives of *T. rubrofasciata*. The gene data obtained in this study will no doubt contribute new evidence for this analysis.

## Conclusions

This is the first record of the presence of a triatomine species in Foshan, Guangdong Province, China. Obviously, the risk of this vector has been underestimated. Therefore, further studies are needed to determine triatomine biology, genetic variations and distributions, fauna, living habits and their genetics throughout Southern China, especially in Guangdong, Fujian, Hainan, Yunnan provinces and Guangxi Zhuang Autonomous Region, which allegedly contained this species of triatomine thirty years ago.

## Additional file

Additional file 1: Multilingual abstracts in the five official working languages of the United Nations. (PDF 755 kb)

## Abbreviations

16S rRNA: 16S ribosomal RNA
28S rRNA: 28S ribosomal RNA
CytB: Cytochrome b
PCR: Polymerase chain reaction
