# Supplementary material for: First records of Triatoma rubrofasciata (De Geer, 1773) (Hemiptera, Reduviidae) in Foshan, Guangdong Province, Southern China
Source: Infect Dis Poverty. 2017 Aug 15;6:129. doi: 10.1186/s40249-017-0342-y (PMC5557067; doi:10.1186/s40249-017-0342-y)
Supplement: Additional file 1: — Multilingual abstracts in the five official working languages of the United Nations. (PDF 755 kb) [file 40249_2017_342_MOESM1_ESM.pdf]

Translation of the abstract into the five official working languages of the United Nations

السجلات الأولى لـ Triatoma rubrofasciata (دي جير، 1773) (نصفيات الجناح، الرضوفيات) في فوشان بمقاطعة جوانجدونج في جنوب الصين

تشين ليو، يون هاي جوه، يي تشانج، تشنج بن زو، ليانج ليانج تشانج، دان تشو، شياو نونج زو

ملخص
خلفية: البق المقبل، المعروف أيضا باسم البق القاتل، الذي يوجد في جميع أنحاء العالم، وخاصة في أمريكا اللاتينية، معروفة جيدا أنه الناقل الطبيعي الهام لنقل داء المثقبيات الأمريكي، والمسمى أيضا مرض شاجاس. في الصين، عرف نوعين من الفسفس (Triatoma rubrofasciata و T. sinica) عبر التاريخ. منذ تزايد التحركات السكانية وتزايد خطر الانتشار العالمي لمرض شاجاس، أصبح البق المقبل مصدر إزعاج للصحة العامة، بدأنا أنشطة رصد البق المقبل في عام 2016 في جنوب الصين.
الأساليب: تم جمع عينات من البق المقبل من قبل المعهد الوطني للأمراض الطفيلية، المركز الصيني لمكافحة الأمراض والوقاية منها والتي تم تحديدها من خلال خصائصها المورفولوجية تحت مجهر الفحص الدقيق. بالإضافة إلى التحليل المورفولوجي، تم استخراج الحمض النووي الجيني من العينات، والجين الريباسي الميتوكوندري S16 والسيتوكروم ب (CytB) والجين الريباسي النووي S 28 rRNA تم تكبيرها بواسطة تفاعل البوليميرز المتسلسل لتحليل وتأكيد الأنواع وراثيا.
النتائج: جمعت أنثى واحدة وذكر بالغ واحد من حشرة البق من مسكن في المناطق الريفية من مقاطعة شوند، مدينة فوشان بمقاطعة جوانجدون، الصين (22 ° 44.63'42 "N، 113 ° 45.34'08 "E). نتيجة التحليل المورفولوجي وتحليل الجينات أشارت إلى أن هذه الترياتومينات كانت T. rubrofasciata.
الاستنتاجات: تثبت نتائجنا أنA. cantonensis هي النوع الوحيد من فصيلة الديدان الرئوية في الفئران في جمهورية الصين الشعبية ويظهر بها تنوع وراثي واضح. ينبغي أن تكون هناك مزيد من الدراسات للتوصل إلى فهم أوضح لبيئة هذا النوع من البق المقبل، لأنها وجدت مصابة بشكل طبيعي بـ المثقبية الكروزية و T. conorhini كانت هناك أدلة على قدراتها على التوطن.

Translated from English version into Arabic by Mahmoud Sami, through

TRANSLATORS
WITHOUT BORDERS

首次在中国广东佛山记录红带锥蝽（半翅目：猎蝽科）

刘琴，郭云海，张仪，周正斌，张亮亮，朱丹，周晓农

摘要：
引言：锥蝽，又被称作接吻虫，在世界范围内均有发现，特别是在拉丁美洲。锥蝽是美洲锥虫病（又称恰加斯病）的重要媒介。在中国，历史上记载主要有两种锥蝽（红带锥蝽和华锥猎椿）。当前，由于随着人口的流动的增加和恰加斯病全球扩散的风险的增加，从 2016 年起，我们开始在中国南方监测锥蝽。
方法：中国疾病预防控制中心寄生虫病预防控制所收集锥蝽标本，并在解剖显微镜下进行了形态学鉴定。同时，提取了标本的基因组 DNA，并扩增了线粒体基因--16S r 核糖体（16S rRNA）基因和细胞色素 B （CytB）基因以及核基因--28S r 核糖体（28S rRNA）基因，以从基因水平确定物种。

结果：在中国广东省，佛山市，顺德县（22° 42′ 44.63″ N，113° 08′ 45.34″ E）收集到一雌一雄锥蝽成虫。从形态学和基因分析表明这些锥蝽为红带锥蝽（*T. rubrofasciata*）。
结论：这是首次在中国广东省佛山市发现红带锥蝽。我们将进一步的研究以便更清晰的认识这种锥蝽的生态，因为红带锥蝽已经被证实能自然感染克氏锥虫和康氏锥虫，并且克氏锥虫和康氏锥虫能在其体内定殖。

Translated from English version into Chinese by Qin Liu

Premiers signalements de l’espèce *Triatoma rubrofasciata* (De Geer, 1773) (Hémiptères, Réduvidés) à Foshan, dans la province du Guangdong , dans le sud de la Chine

Qin LIU, Yun-Hai GUO, Yi ZHANG, Zheng-Bin ZHOU, Liang-Liang Zhang, Dan ZHU, Xiao-Nong Zhou

Résumé

Contexte: Les Triatomas, également appelés kissing bugs, présents partout dans le monde et particulièrement en Amérique latine, sont bien connus pour avoir agi comme vecteurs naturels importants de la transmission de la trypanosomiase américaine également connue sous le nom de maladie de Chagas. Au cours de l’histoire, la présence de deux espèces de *triatomas*, soit la *Triatoma rubrofasciata* et la *Triatonma sinica*, a été enregistrée en Chine. Depuis l’accroissement des flux de population et la transmission de la maladie de Chagas à l’échelle mondiale, les triatomas sont devenus des nuisances potentielles de la santé publique; nous avons commencé à surveiller les activités des triatomas en 2016, dans le sud de la Chine.
Méthodes: Des spécimens de triatomas ont été recueillis par le National Institute of Parasitic Diseases, Centre chinois pour le contrôle et la prévention des maladies, et ont été identifiés en déterminant leurs caractéristiques morphologiques au moyen du microscope à dissection. En plus de l’analyse morphologique, l’ADN génomique des spécimens en question a été extrait et le gène mitochondrial ARNr 16S et cytochrome b (CytB) ainsi que le gène nucléaire ARN ribosomique 28S ont été amplifiés par PCR afin d’analyser et confirmer génétiquement les espèces.
Résultats: Une femelle et un mâle adulte de l’insecte ont été recueillis dans une habitation de la zone rurale du district de Shunde, de la ville de Foshan, dans la province du Guangdong, en China<e (22°42′44.63″ nord, 113°08′45.34″ est). Le résultat de l’analyse morphologique et génétique indiquait que ces triatomas étaient la *Triatoma rubrofasciata*.
Conclusions: Il s’agit de la première fois que l’apparition de la *Triatoma rubrofasciata* a été constatée à la ville de Foshan, dans la province du Guangdong, dans le sud de la Chine. D’autres études devraient permettre de mieux comprendre l’écologie de cette espèce de triatomas, puisqu’une contamination naturelle par le *Trypanosoma cruzi* et par le *Trypanosoma conorhini* a été constaté et qu’il y a des preuves de la capacité de domiciliation de ces espèces.

Translated from English version into French by Daniele Di Francesco, through

Первые упоминания триатомовых клопов *Triatoma rubrofasciata* (де Гер, 1773) (Полужесткокрылые хищнецы) в Фошане, провинция Гуандунь на юге Китая

Цинь Лю, Юнь-Хай, И Чжан, Чжен-Бинь Чжоу, Лян-Лян Чжан, Дань Чжу, Сяо-Нонь Чжу

Аннотация

Справочная информация: Триатомовые клопы, также известные под названием поцелуйных клопов, распространены по всему миру, особенно на территории Латинской Америки, являясь широко известными переносчиками американского трипаносомоза, также называемого болезнью Шагаса. В истории Китая были зарегистрированы два вида *триатомовых клопов* (*Triatoma rubrofasciata* и *T. sinica*). Вследствие возрастания перемещения населения и, как следствие, увеличения риска распространения болезни Шагаса в глобальном масштабе, триатомовые клопы стали представлять потенциальную опасность для здоровья, и с 2016 года в южном Китае нами проводится наблюдение за их жизнедеятельностью.

Методы: Сбор особей триатомовых клопов был осуществлен Национальным институтом паразитарных болезней, а также Китайским центром по контролю и профилактике заболеваний; определение произведено по морфологическим признакам с использованием стереомикроскопа. Дополнительно к морфологическому анализу было произведено извлечение геномной ДНК образцов, а также в рамках генетического анализа и подтверждения особей были амплифицированы по ПЦР митохондриальная 16S рРНК, ген цитохромы b (CytB), а также и ядерные рибосомные гены 28S рРНК.

Результаты: Две особи насекомых: одной самки и одного взрослого самца, были собраны в жилище сельской местности округа Шуньдэ, города Фошань, провинции Гуандун, Китай (22 ° 42′44. 63″N, 113 ° 08′45. 34″E). Результаты как морфологического, так и генетического анализов указывают на принадлежность данных триатомовых клопов к разновидности *T. rubrofasciata*.

Выводы: Подтверждение присутствия *T. rubrofasciata* в городе Фошане, провинции Гуандун южного Китая было произведено впервые. С целью достижения большей ясности в понимании экологии особей триатомовых клопов требуются дальнейшие исследования, в то время как вследствие обнаружения их естественного заражения *трипаносомой крузи* , а также *T. conorhini* доказуема способность особей к приспособляемости.

Translated from English version into Russian by Liudmila Tomanek (nee Volynets) , through

Primeros registros de *Triatoma rubrofasciata* (De Geer, 1773) (Hemiptera, Reduviidae) en Foshan, provincia de Guangdong, en el sur de China

Qin LIU, Yun-Hai GUO, Yi ZHANG, Zheng-Bin ZHOU, Liang-Liang Zhang, Dan ZHU, Xiao-Nong Zhou

Resumen

Información de referencia: Los triatominos, que se encuentran por todo el mundo, especialmente en América Latina, son bien conocidos como vectores naturales de importancia para transmitir la tripanosomiasis americana, también llamada enfermedad de Chagas. En China se han registrado a lo largo de la historia dos especies de *Triatoma* (*Triatoma rubrofasciata* y *T. sinica*). Debido a los crecientes movimientos de población y al creciente riesgo de difusión global de la enfermedad de Chagas, los triatominos se convirtieron en un potencial problema para la salud pública, iniciamos la supervisión de las actividades de los triatominos en 2016 en el sur de China.

Métodos: Los especímenes de triatominos fueron recogidos por el Instituto Nacional de Enfermedades Parasitarias del Centro para Control y Prevención de Enfermedades de China y se identificaron por sus características morfológicas bajo el microscopio de disección. Además del análisis morfológico, se extrajo el ADN genómico de los especímenes y amplificaron por PCR el 16S rRNA mitocondrial y el gen citocromo b (CytB) así como el 28S rRNA del ribosoma nuclear para analizar y confirmar las especies genéticamente.

Resultados: Se recogieron una mujer y un adulto macho del insecto en una vivienda situada en las zonas rurales del condado de Shunde, ciudad de Foshan, provincia de Guangdong, China (22 ° 42'44.63 "N, 113 ° 08'45.34" E). Resultado de la morfología y el gen Los análisis indicaron que estos triatominos eran *T. rubrofasciata*.

Conclusiones: Es la primera vez que se confirma la *T. rubrofasciata* En la ciudad de Foshan, provincia de Guangdong, en el sur de China. Estudios posteriores deben llegar a una comprensión más clara de la ecología de esta especie de triatominos ya que se ha encontrado naturalmente infectado por *Trypanosoma cruzi* y *T. conorhini* y hay evidencias de sus capacidades de domiciliación.

Translated from English version into Spanish by SergioLorenzi, through

TRANSLATORS
WITHOUT BORDERS
